# Exploring climate-induced sex-based differences in aquatic and terrestrial ecosystems to mitigate biodiversity loss

**DOI:** 10.1038/s41467-023-40316-8

**Published:** 2023-08-16

**Authors:** Elena Gissi, Londa Schiebinger, Elizabeth A. Hadly, Larry B. Crowder, Rosalia Santoleri, Fiorenza Micheli

**Affiliations:** 1 Oceans Department, Hopkins Marine Station, Stanford University, 120 Ocean View Blvd, Pacific Grove, CA 93950 USA; 2 National Research Council, Institute of Marine Science, CNR ISMAR, Arsenale, Tesa 104 - Castello 2737/F, 30122 Venice, Italy; 3 National Biodiversity Future Center, Palermo, 90133 Italy; 4 History of Science, Gendered Innovations in Science, Health & Medicine, Engineering and Environment, Stanford University, Stanford, CA 94305 USA; 5 Department of Biology, Stanford University, Stanford, 94305 CA USA; 6 Stanford Woods Institute for The Environment, Stanford University, Stanford, 94305 CA USA; 7 Center for Innovation in Global Health, Stanford University, Stanford, 94305 CA USA; 8 Stanford Center for Ocean Solutions, 120 Ocean View Blvd, Pacific Grove, CA 93950 USA

**Keywords:** Phenology, Conservation biology

## Abstract

The response of aquatic and terrestrial organisms to climate change can depend on biological sex. A key challenge is to unravel the interactive effects of sex and climate change at the individual and population levels and the cascading effects on communities. This new understanding is essential to improve climate adaptation and mitigation strategies.

Challenges posed by climate change threaten ecosystems, the crucial benefits they provide to people, and, ultimately, human livelihoods and lives. Global change impacts ecological systems and processes across continents and oceans, resulting in changes to the phenology, distribution and behavior of organisms, persistence and dynamics of populations, and structure and function of species assemblages and ecosystems. In the Anthropocene, a deeper understanding of the biological impacts of climate change, as well as innovative solutions, are needed to reverse climate-induced biodiversity loss.

One largely ignored dimension in research and conservation is how organisms respond differently to climate change depending on biological sex^1,2^. Sex is frequently not reported or tested, likely because researchers are not aware of sex-based differences, assume that including sex-specific information would not influence experimental results or are unable to obtain sex-specific data^3^. Here, we discuss examples of sex-based differences across aquatic and terrestrial domains. We argue that studying sex-based differences from organisms to populations and communities may generate new insights into resilience and vulnerability to climate stressors that may otherwise not be recognized.

## Sex influences the responses of organisms, populations, and communities to climate change

### Organisms

**Fig. 1:**
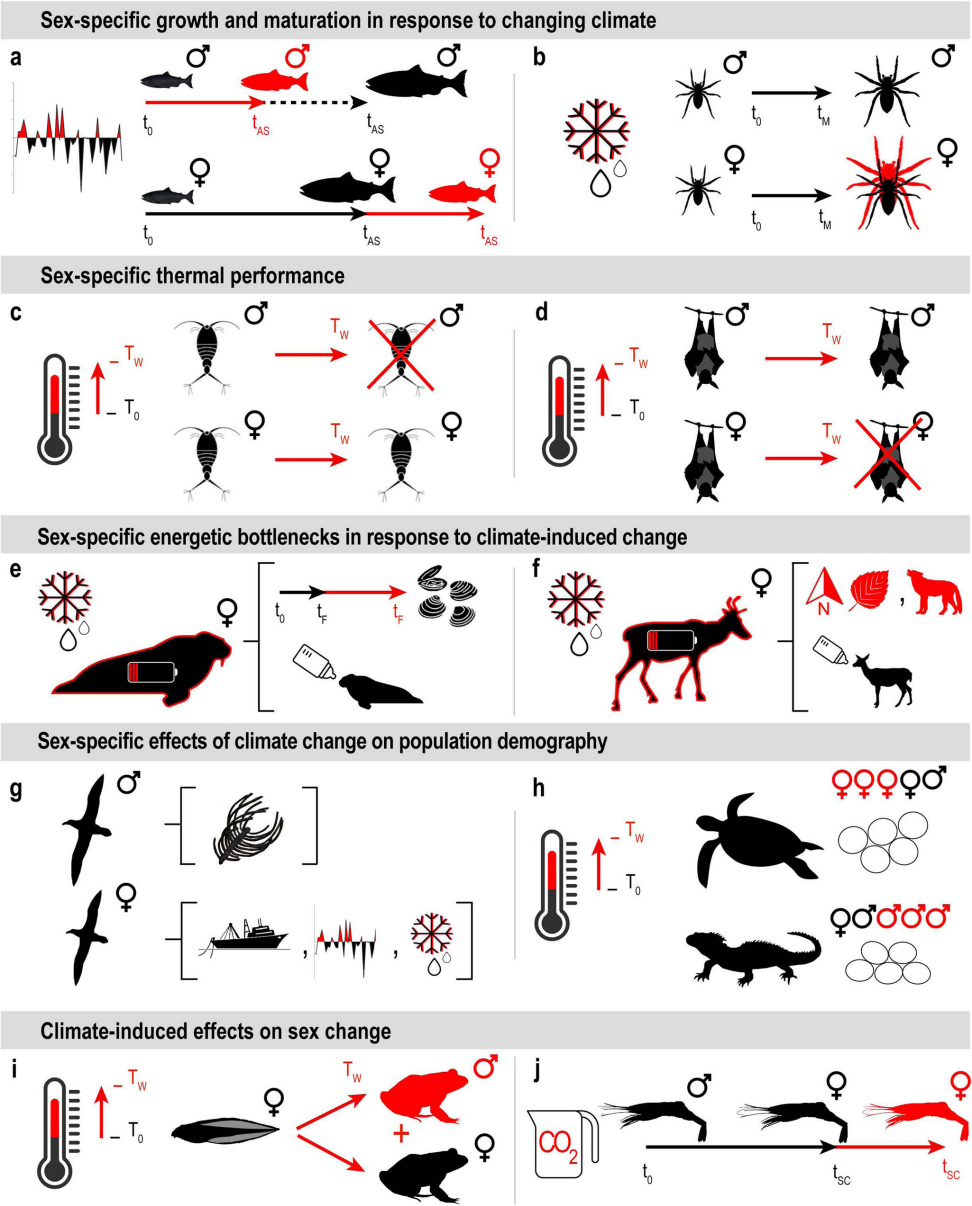
Examples of sex-based responses to climate change at different levels of biological organization. **a** Sex-specific growth and maturation of chum salmon populations to climate regime shift (represented through an oscillating graph). **b** Sex-specific growth and maturation of Arctic wolf spiders to earlier snowmelt (represented through a melting snowflake). Sex-specific thermal performance of copepods (**c**) and flying foxes (**d**) in response to thermal extremes (represented through a thermometer). **e** Sex-specific negative energy balance in female Pacific walruses (represented through a low battery over a full black silhouette with a red outline) resulting from increased time active in water for foraging and sustaining lactation (a baby walrus with a milk bottle) in response to climate-induced sea-ice reduction (a melting snowflake). **f** Sex-specific foraging strategy of caribou facing climate-induced snow cover reduction (represented through a melting snowflake), with females migrating northward toward high-quality feed (a red North sign with a red leaf) and consequently facing higher predation (a red wolf) to sustain lactation (a baby caribou with a milk bottle). **g** Sex-specific effects of fisheries and climate on the demography of sexually dimorphic birds, with males more sensitive to land-based carrions (represented through bones), and females more sensitive to increases in fishing effort (a fishing boat), variation in oceanographic conditions (an oscillating graph) and sea-ice concentration (a melting snowflake). **h** Sex ratio of the hatchlings (eggs represented through oval shapes) altered by temperature change in reptiles. **i** High temperature-induced sex reversal in amphibians. **j** Shift in the timing of sex change in protandrous hermaphroditic shrimp induced by ocean acidification. In the panels, pre-existing conditions or processes not altered by climate change are represented through full black silhouettes and icons. Climate-induced sex-specific changes in species or in conditions encountered by species are represented through red icons, arrows, or letters. T_w_ = temperature increase, t_AS_ = age at spawning, t_M_ = age at maturity, t_F_ = time spent foraging, t_SC_ = time for sex change. Details are reported in Supplementary Table 1. Icons for copepods (author: Phạm Thanh Lộc) and sea turtles (author: Bakunetsu Kaito) were modified from thenounproject.com.

Sex-specific responses to climate change have been documented across a broad range of ecosystems and taxa, from plants to invertebrates, fish, birds and mammals^4^. The broad range of sex-specific responses to different climate stressors is illustrated through selected examples in Fig. 1 and described in more detail below and in Supplementary Table 1.

At the organismal level, individuals can display sex-specific responses to climate stressors that affect body size, growth rates, survival, age or size at first reproduction, and other life-history traits that influence reproductive output and population dynamics and persistence. For instance, the 1988/9 climate regime shift—involving changes in the Pacific Decadal Oscillation Index, the Aleutian Low Pressure Index, sea surface temperature, and zooplankton biomass—triggered sex-specific changes in life-history traits of chum salmon (*Oncorhynchus keta*) populations in the North Pacific^5^ (Fig. 1a). Males and females displayed different maturation schedule and growth in response to the climate shift, with a decrease in body size at spawning for both males and females but an increase in age at spawning for females and a decrease for males. By contrast, females of Arctic wolf spiders (*Pardosa glacialis*) were found to increase in adult body size due to earlier snowmelt more than males^6^ (Fig. 1b). These changes likely decrease reproductive success in chum salmon^5^ and increase reproductive success in Arctic wolf spiders^6^.

### Populations

Monitoring pre-existing sex ratios and exploring factors governing sex ratio mechanisms in natural populations in combination with climate-induced sex-specific effects is essential. It is important to understand the downstream effects of sex in relation to climate stressors on population growth compared to what would be expected if the effect were averaged across the two sexes. In species with temperature-dependent sex determination, an increase in temperature experienced during embryonic development leads, for instance, to more females than males in hatchling sex ratios in green sea turtles (*Chelonia mydas*)^7^ and more males than females in tuatara (*Sphenodon guntheri*)^8^ (Fig. 1h).

Population sex ratio can also change as an effect of different thermal performance between males and females. When exposed to increasing temperatures, for instance, in two populations of Atlantic marine copepods (*Acartia tonsa*), males showed significantly lower survival than females^9^ (Fig. 1c). Reduced reproductive success resulting from greater male mortality and therefore sperm limitation may lead to population decline^9^. Since copulation is necessary for the production of viable eggs, any factor that skews sex ratios toward females and away from males might adversely affect population growth rates through mating limitation^10^. In natural copepod populations, female-skewed ratios are often observed^11^. Under a scenario of increasing temperature, climate-induced male mortality in pre-existing female-skewed copepod populations could affect male-female encounter rates and mating success over time more severely than if thermal performance was similar across sexes. In some species, such as copepods, males showed lower survival than females in response to temperature extremes; in other species, however, such as the Australian flying fox, females showed significantly lower survival than males^12^ (Fig. 1d). Uneven thermal tolerance by males and females causing severe sex ratio distortion in natural populations can affect reproductive success and population viability. Skewed sex ratios can also increase mating competition, decrease mating success, limit fertilization rates and breeding opportunities, and decrease genetic diversity—all of which impact population dynamics and growth^13^. In addition, changes in the fertility of males and females induced by climate change can reduce reproductive performance, worsening the effects on populations in combination with other sex-specific effects. For example, a decrease in the production and viability of sperm was seen in a model beetle species in response to heatwaves, nearly resulting in sterility during successive heatwaves^14^.

Females and males tend to respond differently to climate-induced environmental changes in species where females and males have different energetic requirements, foraging behaviors or habitat preferences (Fig. 1e–g). Consequently, sex-specific sub-population responses to climate change can impact meta-population dynamics by disrupting interactions between sexes. For instance, male and female Pacific walruses (*Odobenus rosmarus divergens*) segregate spatially in the Arctic, with females depending on sea-ice cover for breeding and nursery more than males. A climate-induced reduction in sea ice in the Chukchi Sea forced females to use terrestrial haul-outs instead of sea-ice sites to rest and nurse while foraging in offshore grounds^15^ (Fig. 1e). This situation has increased the time in the water required to reach foraging grounds, which may deplete energy, substantially decrease lactation and limit offspring nutrition, potentially reducing juvenile survival^15^. These climate-induced changes can negatively impact reproductive capacity, population persistence and growth^16^; increased mortality has already been observed as a consequence of juveniles being trampled in terrestrial haul-out sites^17^. Similarly, male and female caribou (*Rangifer tarandus*) segregate spatially following different strategies to maximize reproductive success: males to maximize pre-rut energy reserves and promote successful competition with other males for mates, and females to promote offspring growth and survival^18^. A reduction in high-quality forage owing to climate change means female caribou are likely to move northward to support breeding and lactation, which exposes them to greater predation during migration^19^ (Fig. 1f).

Sex-specific ecological differences can also lead to divergent responses between sexes to climate-induced changes combined with other environmental drivers that negatively affect males and females at different paces. For example, in the case of sexually size-dimorphic seabird populations of Northern and Southern giant petrels (*Macronectes halli* and *M. giganteus*, respectively) in the South Atlantic (Fig. 1g), females, which are smaller than males, rely on pelagic resources for foraging more than males and are more sensitive to increases in variation in oceanographic conditions, sea-ice concentration and fishing effort through entanglements^20^. Females also benefit from stronger meridional winds likely because increased flight speed improves foraging performance. Males, by contrast, rely on terrestrial foraging more than females and are more sensitive to changes in the availability of land-based carrion. Males are also negatively affected by stronger meridional winds likely because of indirect effects from wind-driven changes in oceanographic conditions^20^.

Ignoring sex-based differences leads to underestimates of the relative influence of a changing environment on aquatic and terrestrial populations. More critically, pooling sex-specific data may result in not detecting any effect of environmental drivers or in overestimating effects at a population level. In the case of Northern giant petrels, survival effects were not apparent when females and males were considered together but evident when considered separately^20^. Similarly, in a case of snowy plovers (*Charadrius nivosus*), population viability was significantly overestimated when sex ratio and mating system were ignored^21^, and in a theoretical model of a two-sex spreading population, population invasion was overestimated when sex-specific differences in demographic and dispersal parameters, and in mate-locating mechanisms were overlooked^22^.

### Communities

Sex-specific responses at the species and population levels may impact community dynamics and energy transfer throughout food webs, with consequences for the benefits that humans derive from ecosystems, as seen in the example of Arctic Northern shrimp and related Greenland shrimp fishery production (Fig. 1j). Northern shrimp (*Pandalus borealis*) are protandric hermaphrodites functioning first as males, then transitioning to become females. Ocean acidification and warmer temperatures slow Northern shrimp development such that their transition from male to female no longer coincides with the spring phytoplankton bloom, the major food source for larvae^23^. This situation prolongs larval vulnerability to predation, with negative impacts on recruitment rates. The Greenland shrimp fishery relies on Arctic Northern shrimp stock availability^24^. These climate-induced changes might underlie the decreased shrimp catch rates in this region and the subsequent effects on local employment and economies where fishery processing facilities have already been closing due to a decrease in catch rates since 2012^24^. To fully understand biological responses to climate change, community-level consequences of sex-specific differences at individual and population levels need to be investigated for the higher-order patterns they reveal^25^ (see more examples in Supplementary Table 2). Climate change predictions that assume all individuals of a species are phenotypically identical^26^ may miss critical sex-based intraspecific variability.

## Taking action to reverse biodiversity loss

Sex influences the response of living organisms to climate change in numerous ways. Decision-makers, researchers and funding agencies can act to use this knowledge and produce new insights to advance the fields of global change biology to reverse biodiversity loss (Box 1).

Decision-makers, such as governments or conservation managers, can improve adaptation and mitigation policies by leveraging about sex-specific responses to climate change. For example, to counter the impacts of increasing sand temperatures negatively affecting hatchling sex ratio and hatching success of eggs in six species of marine turtles, the Queensland Government in Australia^27^ devised a conservation strategy to implement practical nest cooling techniques, such as nest shading or egg relocation (Supplementary Box 1). The aim is to increase the production of male hatchlings to achieve ecologically appropriate sex ratios in the Great Barrier Reef populations and to help recover Queensland’s marine turtle stocks in the long term. Although this will be challenging at large spatial scales, the reduced recruitment of sub-adult males could cause catastrophic population declines within one generation, invalidating more than 30 years of marine turtle conservation efforts.

Decision-makers can also consider including sex-specific science of the response of species and ecosystems to climate change in prioritization criteria for new Marine Protected Areas (MPAs). For example, MPAs may not successfully support population viability if they unintentionally protect only one sex of a species, such as Black-vented Shearwaters (*Puffinus opisthomelas*)^28^ that segregate by sex in warmer years (example in Box 1). Research and monitoring are needed to understand how sex-based differences may influence population distribution and dynamics, as well as threats and conservation or management across species and ecosystems.

Research can address the many unanswered questions about biological mechanisms influenced by sex and climate change across species and systems. Researchers have begun to show that climate change can induce sex-specific responses both in the presence and absence of pre-existing sex-specific physiological, metabolic or behavioral differences. Researchers now need to consistently disentangle the interactions between sex and climate stressors to better understand their relative contribution in the response to climate change. This aim will require specific research methodologies able to test sex versus climate change effects independently and in combination.

Moreover, researchers need to focus on how sex-specific responses to climate change at the individual level can propagate across ecosystems, affecting populations and community interactions. Limited research has focused on community-level consequences of sex-specific differences. To advance global change biology, researchers will need to include sex in their experimental approaches whenever feasible. In laboratory experiments, sex-based differences have been tested in less than 10% of the ecological studies reviewed^1,2^, and methodological and logistical challenges constrain sex consideration in field research^4^. Scaling-up results from the laboratory to the natural environment is not straightforward, and results about sex-specific differences from laboratory and field experiments can differ^2^. Nonetheless, results from controlled lab experiments may help identify sex-specific differences and their consequences at population and community levels to be further investigated in natural environments.

Funding agencies can incentivize research aimed at producing sex-specific life-history knowledge for organisms, populations and communities, which will help overcome persistent taxonomic and geographical bias and gaps in biological research^29^ with respect to sex-based differences^4^. This goal can be achieved through targeted funding schemes that support basic research for analysis of sex-related intraspecific variation. Similarly, public institutions and agencies that lead monitoring programs can include sex as a monitoring variable whenever possible to support the acquisition of data disaggregated by sex. Obtaining this “deep peripheral knowledge”^30^—the fine detail of biodiversity needed at the species level—can lead to new research questions in global change biology at population and community levels.

Aquatic and terrestrial biological sciences must more comprehensively and consistently analyze males, females and hermaphrodites across their life and reproductive cycles. Individual sex-specific variability in biological mechanisms induced by climate stressors can cascade from organisms to populations and communities. In addition to the intrinsic value of sex analysis for a deeper understanding of biological systems^4^, the consequences of overlooking sex-based differences can be profound and far-reaching. In particular, efforts to advance climate change adaptation and mitigation for reversing biodiversity loss could fail if sex-specific responses are not considered when designing climate policies and management actions. By coordinating such efforts, decision-makers, researchers and funding agencies can forge a deeper understanding of the biological impacts of climate change that, in turn, can lead to innovative solutions. The sex-specific science of individuals, populations and communities is crucial to safeguarding them and the ecosystems on which they and humanity depend.

Box 1 **Priorities for advancing the field of global change biology toward incorporating sex** As an example, we illustrate the identification of a new Marine Protected Area (MPA) considering species distribution not including (map a) and including knowledge about climate-induced sexual segregation (map b).

*Decision-makers*

- Revising spatial prioritization criteria for new protected areas in relation to sex-specific knowledge on the response of species and ecosystems to climate change;
- Coordinating management and monitoring in relation to sex-specific knowledge for climate adaptation and mitigation.

*Researchers*

- Disentangling the interaction between sex and climate drivers to understand their relative contribution and effects.
- Understanding how sex-specific responses to climate change at an individual level will propagate across systems affecting populations and community interactions.
- Considering sex and sex-based differences in laboratory and in field experiments across species and systems.

*Funding agencies*

- Elaborating funding schemes to support basic research on sex-based differences in species in response to climate change.

***Example of the identification of a new MPA considering sex-specific responses to a changing climate***. Some species, such as Black-vented Shearwaters (*Puffinus opisthomelas*) in coastal waters off Baja California, Mexico^28^, segregate by sex for foraging in warmer years. Sexual segregation only in warmer years might be a foraging strategy during years characterized by lower productivity, leading to sex-specific interannual variation in foraging areas. If a new MPA (in red) is selected without this information (map a), in a warming scenario, the new MPA would unintentionally protect foraging grounds for one sex but not for the other (here, for males but not for females, map b), and consequently impact population dynamics and viability.
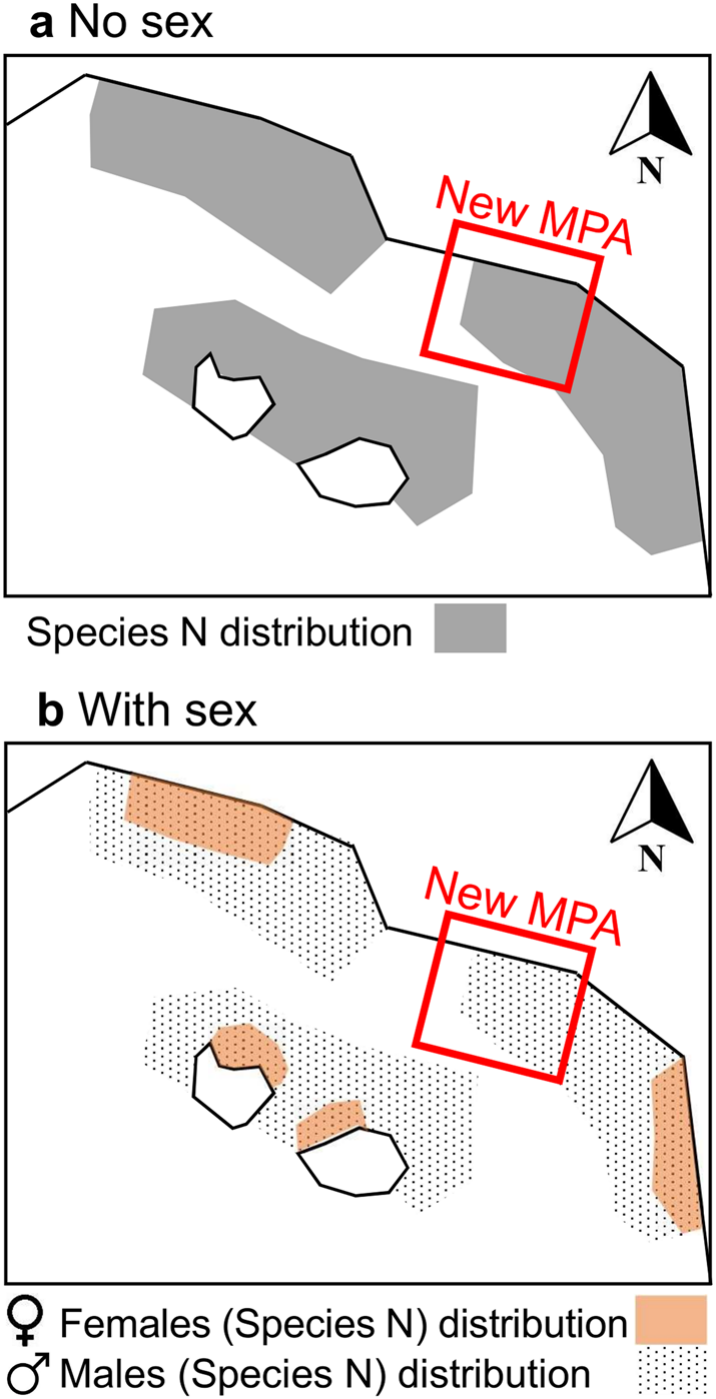

### Supplementary information

Supplementary Information
