# Supplementary material for: Exploring climate-induced sex-based differences in aquatic and terrestrial ecosystems to mitigate biodiversity loss
Source: Nat Commun. 2023 Aug 16;14:4787. doi: 10.1038/s41467-023-40316-8 (PMC10432542; doi:10.1038/s41467-023-40316-8)
Supplement: Supplementary file 1 — Supplementary Information [file 41467_2023_40316_MOESM1_ESM.pdf]

Supplementary Information

Exploring climate-induced sex-based differences in aquatic and terrestrial ecosystems to mitigate biodiversity loss

Elena Gissi[1,2,3], Londa Schiebinger[4], Elizabeth A. Hadly[5,6,7], Larry B. Crowder[1,6], Rosalia Santoleri[2], Fiorenza Micheli[1,6,8]

[1] Oceans Department, Hopkins Marine Station, Stanford University, 120 Ocean View Blvd, Pacific Grove, CA 93950 USA

[2] National Research Council, Institute of Marine Science, CNR ISMAR, Arsenale, Tesa 104 - Castello 2737/F, 30122, Venice, Italy

[3] National Biodiversity Future Center, Palermo 90133, Italy

[4] History of Science, Gendered Innovations in Science, Health & Medicine, Engineering and Environment, Stanford University, Stanford, CA, 94305 USA.

[5] Department of Biology, Stanford University, Stanford, 94305, CA, United States

[6] Stanford Woods Institute for The Environment, Stanford University, Stanford, 94305, CA, USA

[7] Center for Innovation in Global Health, Stanford University, Stanford, 94305, CA, USA

[8] Stanford Center for Ocean Solutions, 120 Ocean View Blvd, Pacific Grove, CA 93950 USA

Supplementary Table 1. Examples of sex-based responses to climate change at different levels of biological organization.

**Sex-specific growth and maturation in response to changing climate**	**Sources**
**a. Sex-specific growth and maturation of fish populations in response to changing climate.** 1988/9 climate regime shift had pervasive effects on female and male maturation schedules and growth in Korean chum salmon (*Oncorhynchus keta*) population, with different maturation trends between males and females, a decrease in body size and an increase in age at spawning in females. These changes have affected the reproductive success of spawning males and females.	Ref.[1]
**b. Sex-specific growth and maturation of Arctic wolf spiders**. Climate change is advancing the onset of the growing season and this is happening at a particularly fast rate in the High Arctic. However, in most species the relative fitness implications for males and females remain elusive. By studying data on 10 successive cohorts of the wolf spider *Pardosa glacialis* from Zackenberg in High-Arctic, northeast Greenland, Høye et al.[2] found marked inter-annual variation in adult body size (carapace width) and this variation was greater in females than in males. Earlier snowmelt during both years of its biennial maturation resulted in larger adult body sizes and a skew towards positive sexual size dimorphism (females bigger than males). These results illustrate the pervasive influence of climate on key life-history traits and indicate that male and female responses to climate should be investigated separately whenever possible.	Refs.[2,3]
**Sex-specific thermal performance**	
**c. Sex-specific thermal performance of copepods in response to climate change.** In two populations of copepods *Acartia tonsa* from Connecticut and Florida (US), males showed significantly lower survival than females when exposed to increasing temperatures. Ignoring sex-specific differences in thermal tolerance may lead to underestimating population decline due to sperm limitation.	Ref.[4]

**d. Sex-specific thermal performance of flying foxes in response to thermal extremes**. Welbergen et al.[5] examined the effects of temperature extremes on behaviour and demography of vulnerable wild flying-foxes (*Pteropus spp.*) in New South Wales, Australia. In 2002 temperatures exceeding 42°C killed over 3500 individuals in nine mixed-species colonies. Young and adult females were more affected than adult males (young, 23–49%; females, 10–15%; males, less than 3%). Such differences have been reported to cause severe sex ratio distortion in some natural populations, such as in tropical black flying foxes where 84% of adults killed by an extreme high-temperature event were females.	Ref.[5]
**Sex-specific energetic bottlenecks in response to climate-induced change**	
**e. Sex-specific caloric intake of Pacific walruses in response to climate-induced sea ice reduction.** Increased the time active in water may result in negative energy balance in female walruses (*Odobenus rosmarus divergens*) that precludes them from sustaining lactation, impacting their capacity to reproduce, and limiting adequate nutrition for offspring, potentially reducing juvenile survival - a development that may impact overall walrus population demographics and survival. Females rely on the presence of sea ice to give birth and nurse their calves. They migrate annually to the ice edge through the fall, they breed in winter and return to the Bering Sea in spring. On the contrary, males are less dependent on sea-ice cover. Their haul-out locations are prominent throughout the year along ice-free portions of the Bearing Sea and in the southeast Bering Sea in open water conditions. As a consequence of the shift to terrestrial haul-outs and of the likely increase of exposure to anthropogenic stressors, an unusually high mortality rate was observed in walruses in coastal Alaska and Russia in 2007 and 2009[6] especially of juveniles being trampled[7]. The autumn retreat of sea ice to the north of the continental shelf has become a common occurrence, and so the use of terrestrial haul-outs for females.	Refs.[8–12], [13,14]
**f. Sex-specific foraging strategy of Caribous facing climate-induced snow cover reduction.** Caribous experience nutritional deficiencies in the calving season. Lactating females have higher daily food intake, forage longer than non-reproductive females, and prioritize quality (i.e., high nitrogen) feed. Foraging strategies of lactating females will be probably altered facing climate change. Females can migrate northward to enhance high quality forage but increase the risk of predation. As an alternative strategy, females can forage longer to compensate for the decrease of forage quality but increasing insect harassment in warmer climate. Overall, reduced forage quality might have a negative impact on caribou fitness and population growth.	Refs.[15,16]
**Sex-specific effects of climate change on population demography**	
**g. Sex-specific effects of fisheries and climate on the demography of sexually dimorphic birds.** In Southern Atlantinc northern (*Macronectes halli*) and southern (*M. giganteus*) giant petrels females are more sensitive to increases in fishing effort, variation in oceanographic conditions and sea ice concentration. Males, by contrast, are more sensitive to land-based carrions. Ignoring sex-based differences could underestimate the relative influence of a changing environment on population survival and reduce the effectiveness of conservation strategies.	Ref.[17]
**h. Sex ratio altered by temperature change in reptiles.** Changes in environmental temperature can profoundly alter the sex ratio of temperature-dependent sex determination species. Environmental conditions experienced during embryonic development have population-scale consequences in taxa for which offspring sex is irreversibly determined by thermal regimes experienced during development[18], such as reptiles[18], amphibians[19], and birds[20]. Equal numbers of male and female offspring are produced at a pivotal temperature, whereas higher or lower ambient temperatures result in consistent sex ratio bias[20] with increasing temperatures leading to more females in hatchling sex ratios in sea turtles[21–23] and more males in tuatara (*Sphenodon guntheri*)[24]. Understanding sex ratios is indeed essential for conservation management effectiveness. In the case of sea turtles, knowledge of females through flipper tagging and satellite tracking studies is abundant since females predictably appear on nesting beaches where researchers can tag them[23,25,26]. Knowledge of male turtles movement, by contrast, is limited as males rarely come on-shore and tagging them offshore is difficult and costly[23,27–29]. Knowing more about male sea turtles is essential, particularly in light of the rapid increase in females in sea turtle populations due to global warming[22,23].	(various)
**Effects on sex change**	
**i. High temperature-induced sex reversal in amphibians.** Temperature-induced sex reversal refers to cases in which sex is initially determined genetically, but is then altered by environmental temperature[30]. Mikò et al.[31] found female-to-male sex-reversing effects of high temperature in agile frogs (*Rana dalmatina*). High temperature induced female-to-male sex reversal, decreased survival, delayed	Refs.[30,31]

metamorphosis, decreased body mass at metamorphosis, and increased the proportion of animals that had no fat bodies. climate change and chemical pollution may have complex consequences for individual fitness and population persistence in species with environment-sensitive sex determination. The actual fitness effects of sex reversal are incompletely known. For a review on the topic, see ref.[30].	
**j. Shift in timing of sex change in protandrous hermaphrodites.** Ocean acidification can affect the timing of the transition from male to female in Arctic Northern shrimps (*Pandalus borealis*). This will likely affect sex ratio and may impact Greenland fishery production.	Ref.[32]

Supplementary Table 2. Examples of cascading effects of sex-based response to climate change, from organisms and population to communities.

Case study/species	Male vs female differences	Climate effect	Cascading/population effect	Sources
Interaction of species with habitat: Valerian (*Valeriana edulis*) and arthropods	In valerian (*Valeriana edulis*), a dioecious herb with elevation range from arid low-elevation scrublands to mesic alpine tundra (2000 to 3790 m) of Colorado (USA), female plants were more frequent than males in high elevations because of sex-specific water use efficiency and survival with high water availability.	Because of recent climate-induced aridification, male frequency increased and moved upslope, resulting in greater pollination success and increased seed set for females, facilitating species upslope range expansion.	Since female *V. edulis* support higher densities of arthropods than males, including several specialist herbivores that depend exclusively on *V. edulis*, the effects of a climate-driven decline in female frequency at low elevations may have cascading impacts of associated arthropod communities.	Ref.[33–35]
Predator-prey interaction: American lobster (*Homarus americanus*) and fishery catches	In American lobster (*Homarus americanus*) populations in estuaries, nearshore coastal and offshore habitats of the North-Western Atlantic, sex ratios are skewed towards males in some locations and females in others. Males prefer lower salinity habitats or remain in nearshore and estuarine habitats more than females, which prefer deeper cooler offshore areas. Moreover, fisheries regulations targeting males over reproductive females affect fishing mortality, which depends also on sex-specific catchability and behaviour.	Climate-induced changes in thermal gradients have likely been a key component in the observed changes in distribution patterns.	Projected water temperature increase will likely exacerbate existing skewed sex ratios with effects at local scale, particularly when coupled with sex-specific fishing pressure. These effects will likely influence community diversity and fisheries catches.	Ref.[36]
Host-parasite interaction: Meerkats	In meerkats (*Suricata suricatta)* of Kalahari Desert (Southern Africa),	Temperature extremes affect clinical tuberculosis occurrence especially in	Climate change can affect social groups by increasing infectious disease	Ref.[37]

(*Suricata suricatta)* and tuberculosis infection by *Mycobacterium suricattae*	cooperative breeders living in groups of 2–50 individuals, a type of tuberculosis caused by infection with the species *Mycobacterium suricattae* is endemic and widespread. Females, evicted by the group dominant female during breeding seasons, return to their group. Males emigrate voluntarily over large distances. These population dynamics may decrease group size and increase female/male ratio in groups, increasing the change of immigration of males.	groups that have had a high number of male immigrants in the 5 months preceding the first clinical tuberculosis cases. The probability of group extinction is high with high temperature extremes and male emigration.	outbreaks, i.e., altering dynamics and interactions between hosts and parasites. Males are likely important carriers of tuberculosis because of sex-specific immunological responses.	

Supplementary Box 1. Example of a policy implemented in Australia to counteract the effects of the increase in temperature on sea turtle populations.

The Department of Environment and Science of the State of Queensland, Australia, designed the "Queensland marine turtle conservation strategy 2021–2031"[38] to counter the impacts of increasing sand temperatures negatively impacting hatchling sex ratio and hatching success of eggs in six species of marine turtles. The conservation strategy consists in implementing practical nest cooling techniques, such as nest shading or egg relocation. The aim is to increase the production of male hatchlings to achieve ecologically appropriate sex ratios in the Great Barrier Reef populations, and to help recover Queensland's marine turtle stocks in the long term. Although this will be challenging at large spatial scales, the reduced recruitment of sub-adult males could cause a catastrophic stock decline within one generation, impacting more than 30 years of marine turtle conservation efforts. The plan has established the following Target 3.2.: "Nesting success and survivability of marine turtle clutches of eggs and hatchlings is increased to more than 80% with a target sex ratio above 30% male to support long-term stock recovery (the 80% target is higher than in the national Recovery Plan and considered necessary to recover depleted stocks in the face of increasing threats such as climate change).". To combat this, the department's researchers used shade cloth to keep the sand temperature cool enough for turtle nests. More than 300 clutches of eggs were relocated to shaded areas of the beach in one nesting season. Sand surface temperatures under the shade cloth were around 30° cooler than on the exposed dunes and the department's researchers were confident that at nest depth, which is up to 60cm below the sand, temperatures remained lower than the critical 32°. This simple idea makes a real difference to the numbers of hatchling turtles produced at Mon Repos which is critical to the survival of the loggerhead turtle. More than 1300 clutches of eggs hatch in total each nesting season. Mon Repos is of particular importance as a nesting site for the endangered loggerhead turtle, which lays around 125 eggs per clutch. Green turtles and flatbacks also use the rookery, laying 115 eggs and 50 eggs per clutch, respectively. The conservation strategy includes i) monitoring pivotal temperature – the temperature at which 50% of the hatchlings are female – and nest temperature at local conditions for the different populations and ii) testing management actions effectiveness. Monitoring protocols in marine protected areas have been updated to include sex-specific considerations.

Supplementary References

1. Urbach, D. *et al.* Growth and maturation of Korean chum salmon under changing environmental conditions. *Fish. Res.* **134–136**, 104–112 (2012).
2. Høye, T. T., Hammel, J. U., Fuchs, T. & Toft, S. Climate change and sexual size dimorphism in an Arctic spider. *Biol. Lett.* **5**, 542–544 (2009).
3. Bowden, J. J., Høye, T. T. & Buddle, C. M. Fecundity and sexual size dimorphism of wolf spiders (Araneae: Lycosidae) along an elevational gradient in the Arctic. *Polar Biol.* **36**, 831–836 (2013).
4. Sasaki, M., Hedberg, S., Richardson, K. & Dam, H. G. H. G. Complex interactions between local adaptation, phenotypic plasticity and sex affect vulnerability to warming in a widespread marine copepod. *R. Soc. Open Sci.* **6**, (2019).
5. Welbergen, J. A., Klose, S. M., Markus, N. & Eby, P. Climate change and the effects of temperature extremes on Australian flying-foxes. *Proc. R. Soc. B Biol. Sci.* **275**, 419–425 (2007).
6. Jay, C. V., Marcot, B. G. & Douglas, D. C. Projected status of the Pacific walrus (Odobenus rosmarus divergens) in the twenty-first century. *Polar Biol.* **34**, 1065–1084 (2011).
7. Born, E. W., Wiig, Ø. & Olsen, M. T. Chapter 13 - The future of Atlantic walrus in a rapidly warming Arctic. in *The Atlantic Walrus* (eds. Keighley, X., Olsen, M. T., Jordan, P. & Desjardins, S.) 309–332 (Academic Press, 2021). doi:10.1016/B978-0-12-817430-2.00012-1.
8. Noren, S. R. S. R., Udevitz, M. S. M. S. & Jay, C. V. C. V. Sex-Specific Energetics of pacific walruses (Odobenus rosmarus divergens) during the nursing interval. *Physiol. Biochem. Zool.* **89**, 93–109 (2016).
9. Fay, F. H. Ecology and Biology of the Pacific Walrus, Odobenus rosmarus divergens Illiger. *North Am. Fauna* 1–279 (1982) doi:10.3996/nafa.74.0001.
10. Maccracken, J., Beatty, W., Garlich-Miller, J., Kissling, M. & Snyder, J. *Final Species Status Assessment for the Pacific Walrus (Odobenus rosmarus divergens), May 2017 (Version 1.0)*. (2017). doi:10.13140/RG.2.2.29363.12325.
11. Fischbach, A. S., Taylor, R. L. & Jay, C. V. Regional walrus abundance estimate in the United States Chukchi Sea in autumn. *J. Wildl. Manag.* **86**, e22256 (2022).
12. Jay, C. V., Taylor, R. L., Fischbach, A. S., Udevitz, M. S. & Beatty, W. S. Walrus haul-out and in water activity levels relative to sea ice availability in the Chukchi Sea. *J. Mammal.* **98**, 386–396 (2017).
13. Koch, C. W. *et al.* Female Pacific walruses (Odobenus rosmarus divergens) show greater partitioning of sea ice organic carbon than males: Evidence from ice algae trophic markers. *PLOS ONE* **16**, e0255686 (2021).
14. Udevitz, M. S., Taylor, R. L., Garlich-Miller, J. L., Quakenbush, L. T. & Snyder, J. A. Potential population-level effects of increased haulout-related mortality of Pacific walrus calves. *Polar Biol.* **36**, 291–298 (2013).
15. Dong, J. & Anderson, L. J. Predicted impacts of global change on bottom-up trophic interactions in the plant-ungulate-wolf food chain in boreal forests. *Food Webs* **33**, e00253 (2022).
16. Denryter, K., Cook, R. C., Cook, J. G., Parker, K. L. & Gillingham, M. P. State-dependent foraging by caribou with different nutritional requirements. *J. Mammal.* **101**, 544–557 (2020).
17. Gianuca, D. *et al.* Sex-specific effects of fisheries and climate on the demography of sexually dimorphic seabirds. *J. Anim. Ecol.* **88**, 1366–1378 (2019).
18. Mainwaring, M. C. *et al.* Climate change and nesting behaviour in vertebrates: a review of the ecological threats and potential for adaptive responses. *Biol. Rev.* **92**, 1991–2002 (2017).
19. Eggert, C. Sex determination: the amphibian models. *Reprod. Nutr. Dev.* **44**, 539–549 (2004).
20. Mitchell, N. J. & Janzen, F. J. Temperature-Dependent Sex Determination and Contemporary Climate Change. *Sex. Dev.* **4**, 129–140 (2010).
21. Schwanz, L. E. & Janzen, F. J. Climate change and temperature-dependent sex determination: Can individual plasticity in nesting phenology prevent extreme sex ratios? *Physiol. Biochem. Zool.* **81**, 826–834 (2008).
22. Jensen, M. P. *et al.* Environmental Warming and Feminization of One of the Largest Sea Turtle Populations in the World. *Curr. Biol.* **28**, 154-159.e4 (2018).
23. Schofield, G. *et al.* Long-term photo-id and satellite tracking reveal sex-biased survival linked to movements in an endangered species. *Ecology* **101**, e03027 (2020).

24. Mitchell, N. J., Kearney, M. R., Nelson, N. J. & Porter, W. P. Predicting the fate of a living fossil: how will global warming affect sex determination and hatching phenology in tuatara? *Proc. R. Soc. B Biol. Sci.* **275**, 2185–2193 (2008).
25. Jeffers, V. F. & Godley, B. J. Satellite tracking in sea turtles: How do we find our way to the conservation dividends? *Biol. Conserv.* **199**, 172–184 (2016).
26. Rees, A. F. *et al.* Are we working towards global research priorities for management and conservation of sea turtles? *Endanger. Species Res.* **31**, 337–382 (2016).
27. Fitzsimmons, N. N. *et al.* Philopatry of male marine turtles inferred from mitochondrial DNA markers. *Proc. Natl. Acad. Sci. U. S. A.* **94**, 8912–8917 (1997).
28. Rees, A. F. *et al.* Linking loggerhead locations: using multiple methods to determine the origin of sea turtles in feeding grounds. *Mar. Biol.* **164**, 1–14 (2017).
29. Godley, B. *et al.* Satellite tracking of sea turtles: Where have we been and where do we go next? *Endanger. Species Res.* **4**, 3–22 (2008).
30. Edmands, S. Sex Ratios in a Warming World: Thermal Effects on Sex-Biased Survival, Sex Determination, and Sex Reversal. *J. Hered.* **112**, 155–164 (2021).
31. Mikó, Z. *et al.* Sex reversal and ontogeny under climate change and chemical pollution: are there interactions between the effects of elevated temperature and a xenoestrogen on early development in agile frogs? *Environ. Pollut.* **285**, 117464 (2021).
32. AMAP. *AMAP Assessment 2018: Arctic Ocean Acidification*. (2018).
33. Petry, W. K. *et al.* Sex-specific responses to climate change in plants alter population sex ratio and performance. *Science* **353**, 69–71 (2016).
34. Petry, W. K. *et al.* Mechanisms underlying plant sexual dimorphism in multi-trophic arthropod communities. *Ecology* **94**, 2055–2065 (2013).
35. Etterson, J. R. & Mazer, S. J. How climate change affects plants' sex lives. *Science* **353**, 32–33 (2016).
36. Jury, S. H. H., Pugh, T. L. L., Henninger, H., Carloni, J. T. T. & Watson, W. H. H. Patterns and possible causes of skewed sex ratios in American lobster (Homarus americanus) populations. **63**, 189–199 (2019).
37. Paniw, M. *et al.* Higher temperature extremes exacerbate negative disease effects in a social mammal. *Nat. Clim. Change 2022 123* **12**, 284–290 (2022).
38. Department of Environment and Science. *Queensland Marine Turtle Conservation Strategy (2021-2031). Queensland Government*. (2021).
